# DFT insights into bandgap engineering of lead-free LiMCl_3_ (M = Mg, Be) halide perovskites for optoelectronic device applications

**DOI:** 10.1038/s41598-025-90621-z

**Published:** 2025-02-26

**Authors:** Apon Kumar Datta, M. Khalid Hossain, Md. Shahriar Rahman, Prabhu Paramasivam, Adel El-marghany, V. K. Mishra

**Affiliations:** 1 Department of Electrical and Electronic Engineering, Mymensingh Engineering College, Mymensingh, 2200 Bangladesh; 2 Institute of Electronics, Atomic Energy Research Establishment, Bangladesh Atomic Energy Commission, Dhaka, 1349 Bangladesh; 3 Department of Advanced Energy Engineering Science, Interdisciplinary Graduate School of Engineering Sciences, Kyushu University, Fukuoka, 816-8580 Japan; 4 Department of Electrical and Electronic Engineering, Hajee Mohammad Danesh Science and Technology University, Dinajpur, 5200 Bangladesh; 5 Department of Research and Innovation, Saveetha School of Engineering, SIMATS, Chennai, Tamilnadu 602105 India; 6 Department of Mechanical Engineering, Mattu University, 318 Mettu, Ethiopia; 7 Department of Chemistry, College of Science, King Saud University, P.O. Box 2455, 11451 Riyadh, Saudi Arabia; 8 School of Chemical Engineering, Yeungnam University, Gyeongsan, 38541 Republic of Korea

**Keywords:** DFT, Lead-free perovskite, Hydrostatic pressure, Bandgap engineering, Optical properties, Mechanical properties, Optical physics, Engineering, Physics

## Abstract

In this theoretical analysis, the pressure-dependent structural, electronic, mechanical, and optoelectronic properties of LiMCl_3_ (M = Mg, Be) have been calculated using density functional theory within the framework of the GGA PBE and hybrid HSE06 functional. At ambient pressure, the calculated lattice parameters of LiMCl_3_ match well with previously reported values, validating the accuracy of this study. Geometry optimization reveals that under increasing hydrostatic pressure, both the lattice parameters and the unit cell volume decrease. Additionally, the band structure exhibits notable phenomena over the pressure range from 0 to 100 GPa. For the LiMgCl_3_ compound, the bandgap decreases from an indirect bandgap of 4 eV to a direct bandgap of 2.563 eV. Similarly, LiBeCl_3_ shows an indirect bandgap that decreases from 2.388 eV to 0.096 eV over the pressure range from 0 to 100 GPa. The optical properties of LiMCl_3_, including absorption coefficient, reflectivity, refractive index, dielectric function, and conductivity, have been calculated throughout the study under varying pressure conditions. The analysis reveals that the optical properties of LiMCl_3_ (M = Be, Mg) enhance with increasing hydrostatic pressure, thereby rendering these materials more suitable for optoelectronic applications. To assess the stability of these compounds, elastic constants were analyzed, indicating that LiMCl_3_ exhibits ductile and anisotropic characteristics under different pressure conditions. These investigated materials are suitable for use in optoelectronic devices due to their favorable physical properties under different pressure circumstances.

## Introduction

Perovskites are demonstrating exceptional performance due to their numerous optoelectronic properties^1–9^, making them suitable for various optoelectronic applications, including solar cells^10–18^, photodetectors^19^, light-emitting diodes (LEDs)^19^, lasers^20^, memristors^21^, and artificial synapse devices^22^. Their properties can vary widely, ranging from metallic to semiconducting to conductive^23^, depending on the composition of the different materials used^24,25^. Perovskites are often referred to as the ‘department store of physical properties’ due to their photocatalytic^26^, dielectric^27^, ferroelectric^28^, pyroelectric^29^, piezoelectric^30^, magnetic^31^, superconductivity^32^, and ionic conductivity^33^. Perovskites often have the formula “ABX_3_”, in which “X” is an anion, usually oxygen or a halogen, and “A” and “B” are cations. Making sure that neither the A-site nor the B-site cations contain lead results in the formation of lead-free perovskites. Lead (Pb²⁺) is instead replaced with alternative cations including Sn²⁺, Ge²⁺, Mg²⁺, etc. Lead-free metal halide perovskites are increasingly used in various devices due to their non-toxicity, supporting the green technology revolution^34–36^. These materials also possess outstanding photoelectronic and thermoelectric properties, including a high dielectric constant, a low exciton binding energy, and a high absorption coefficient. These properties allow for efficient electron-hole pair photogeneration, which makes them suitable for using as absorber materials in solar cells as well as thermoelectric applications^1–5,37–40^. Over the past years, lead-free perovskite-based solar cells have demonstrated greater than 20% efficiency both experimentally and in theoretical studies^41,42^. Under light exposure, halide perovskites exhibit effective flexoelectric coefficients that are too much higher than typical dielectric materials^43^. Flexoelectricity involves generating energy through non-uniform deformations of dielectric materials, regardless of their symmetry. Additionally, the use of piezoelectric materials in a wide range of electronic devices is expanding the piezoelectric device market globally on a daily basis. Thus, it is imperative to employ more lead-free perovskites rather than lead perovskites in order to address environmental and health issues. Lead-free perovskites are also increasingly finding applications in energy storage systems including Li-ion and photo rechargeable batteries. Employing lightweight materials for energy conversion and storage renders them exceptionally well-suited for integration into electric vehicles and portable electronic devices^44^.

Numerous techniques including applying pressure^45,46^ and adding dopants^47,48^ can alter the physical properties of materials, rendering them suitable for various optoelectronic devices^36,49^. To increase photocurrent density, Sr is introduced into CsPbBr_3_ perovskite, as reported by Arindam et al.^50^. Linh et al. reported that (Bi_0.5_M_0.5_)TiO_3_’s direct band gap increased when larger ionic radius alkali metals (Li, Na, and K) were substituted^51^. S Kurra et al. reported enhanced photocatalytic activities of Na_0.5_Bi_0.5_TiO_3_ perovskite when it is doped with metal ions (Ag^+^, Cu^2+^, and Sn^2+^). Moreover, in a prior investigation, it was illustrated that the application of hydrostatic pressure leads to a reduction in the bandgap, shifting from the ultraviolet to the visible spectrum. This manipulation also facilitates a transition from an indirect to a direct bandgap, thereby augmenting the material’s viability for optoelectronic applications^52^. M. Aslam et al. also reported a decrease in bandgap and enhanced optoelectronic properties of CdZrO_3_ perovskite under various hydrostatic pressure conditions in their study^53^. Various alkaline earth metal perovskites are increasingly being considered for photovoltaic applications due to their suitable bandgaps and higher absorption coefficients^54,55^. Recent investigations using first-principles DFT calculations have highlighted the potential of alkaline metal-based halide perovskites, such as LiMgCl_3_ and LiBeCl_3_, for applications in energy storage devices, scintillating materials, and various modern technologies^56^. In a previous study, ‘Be’ and ‘Mg’ have a significant impact on the structural, elastic, and optoelectronic characteristics of ternary LiMCl_3_ (R = Mg and Be) perovskites. The bandgap energy of these materials indicates their insulating nature, which is a limit to employ these materials in optoelectronic devices that need comparatively narrow bandgaps for easier transition of electrons. As pressure has been widely studied for its ability to modify the physical properties of various materials, there remains a lack of theoretical or experimental investigations into the effects of pressure on the cubic phase of LiMgCl_3_ and LiBeCl_3_. Therefore, we applied hydrostatic pressure to observe the changes in their physical properties and evaluate their potential viability for optoelectronic applications.

The Generalized Gradient Approximation (GGA) and the Perdew-Burke-Ernzerhof (PBE) exchange-correlation functional have been employed within the CASTEP for this calculation. Mechanical properties are analyzed to assess the stability of the investigated materials. Band structure, total density of states (TDOS), and partial density of states (PDOS) have been calculated across a pressure range of 0 to 100 GPa. Additionally, optical properties such as reflectivity, refractive index, absorption coefficient, and dielectric functions have been evaluated to explore their potential applications in optoelectronic devices. The selection of this pressure range may have been motivated by the goal of achieving better optoelectronic properties compared to ambient pressure, specifically to enhance absorption in both the visible and UV photon energy ranges, with a primary focus on the UV region. This is especially relevant for multilayer solar cells, such as tandem solar cells, where enhanced absorption in the UV region can improve the performance of solar cell devices. Additionally, reducing the bandgap facilitates a smoother transition of electrons from the valence band to the conduction band, which benefits optoelectronic devices. Given these considerations, our study demonstrates that these outcomes are achievable within a pressure range up to 100 GPa, and therefore, further investigation beyond this pressure condition was not pursued.

## Computational details

In this study, First-principles calculations based on Density Functional Theory (DFT)^57^, are implemented through the Cambridge Serial Total Energy Package (CASTEP) code^58,59^. The interactions between ions and electrons are described using ultrasoft pseudopotentials in conjunction with the GGA-PBE exchange-correlation functional^60,61^. For geometry optimization, the Broyden–Fletcher–Goldfarb–Shanno (BFGS) technique has been employed^62^, while electronic structure calculations are conducted using density mixing. Geometry optimization involves relaxing the unit cell structures while computational physical parameters are determined under conditions of zero applied pressure and a temperature of 0 K. The plane wave cut-off energy has been set as 550 eV where For Brillouin zone sampling, the Monkhorst-Pack method^62^ is employed with a 5 × 5 × 5 k-point mesh. This ensures sufficient convergence of both the overall energy and the geometrical configuration. The convergence criteria are defined by the following parameters: a maximum ionic Hellmann-Feynman force of 0.01 eV/Å, a maximum displacement of 5 × 10⁻⁴ Å, a maximum stress of 0.02 GPa, and a total energy difference within 5 × 10⁻⁶ eV per atom. For visualizing the optimized structures, VESTA software has been used in this study^63^. The “finite-strain” approach included in the CASTEP code is used to obtain the elastic constants^64^. The ELATE program^65^ is then utilized to generate three-dimensional mechanical anisotropic contour plots for various key mechanical parameters, such as Young’s modulus (E), shear modulus (G), and Poisson’s ratio (ν).

## Results and discussion

### Structural properties

**Fig. 1:**
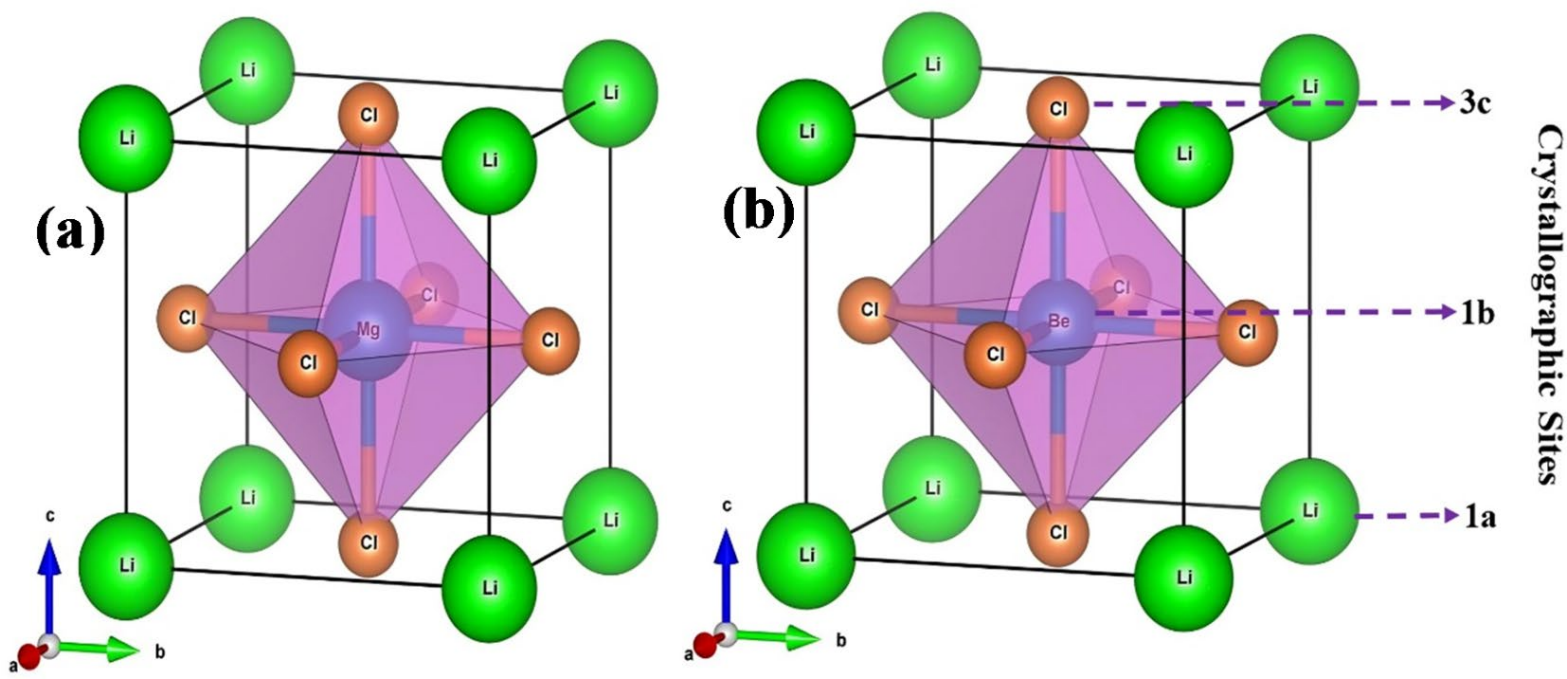
Crystal structure of cubic (**a**) LiMgCl_3_ and (**b**) LiBeCl_3_.

**Fig. 2:**
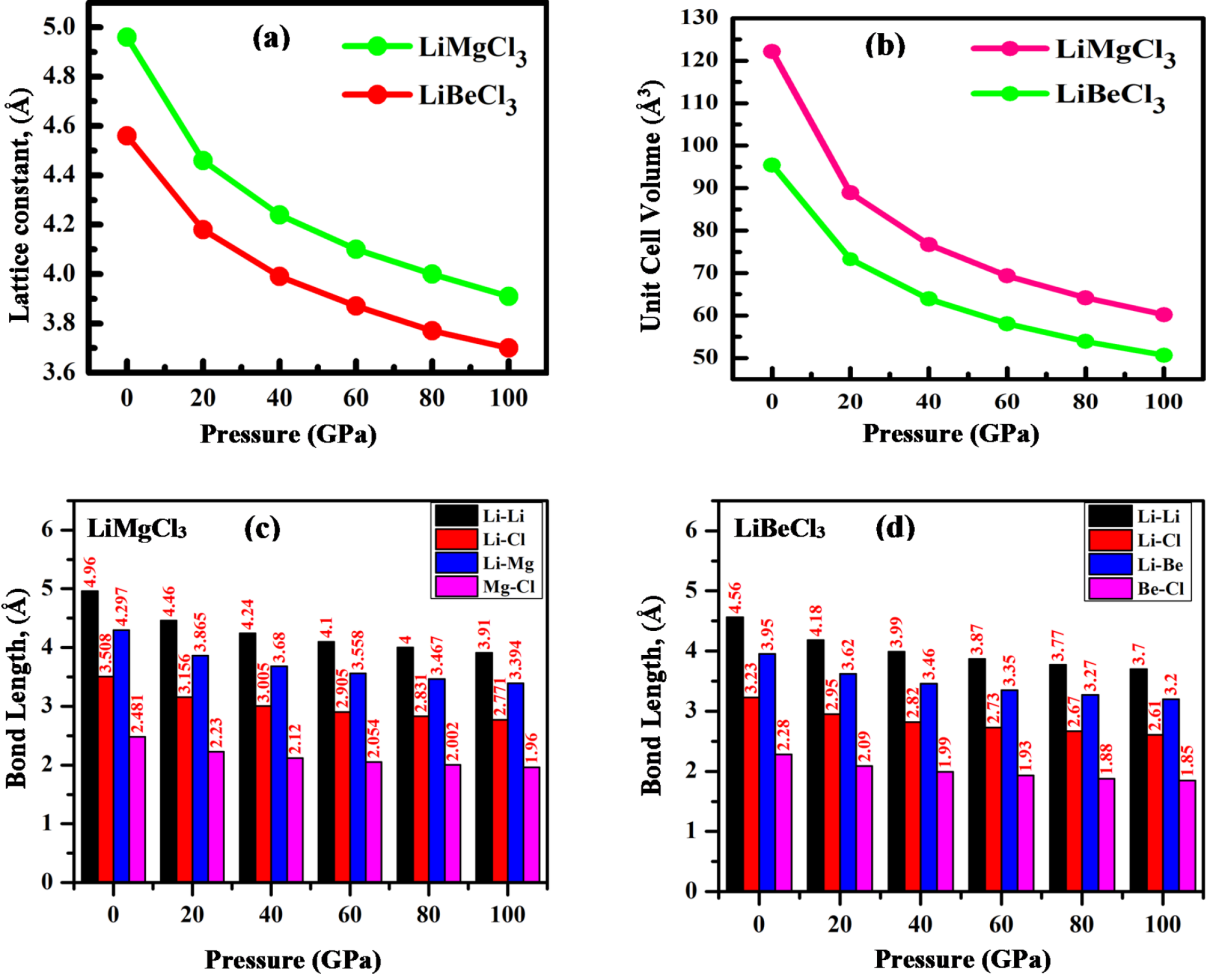
Pressure-induced variation of (**a**) lattice parameters, (**b**) unit cell volume of LiMCl_3_, and variation on bond length of (**c**) LiMgCl_3_ and (**d**) LiBeCl_3_.

The cubic perovskite-type structure of lead-free halide LiMCl_3_ (M = Mg, Be) crystallizes with the space group Pm3̅m (#221). Fig. 1 illustrates that each unit cell of cubic LiMCl_3_ (M = Mg, Be) perovskites contains five atoms: Li occupies the corner position with the Wyckoff position 1a (0,0,0), Mg (Be) located at the body-centered position 1b (0.5, 0.5, 0.5), and Cl atoms are positioned at the face-centered positions with the Wyckoff position 3c (0, 0.5, 0.5). This study analyzes the pressure-induced structural properties of LiMCl_3_ compounds through geometry optimization under various hydrostatic pressures. At ambient pressure, the lattice parameters for LiMgCl_3_ and LiBeCl_3_ compounds have been calculated as 4.96 Å and 4.56 Å, respectively. These values closely align with previously reported data^57^, validating the accuracy of this study using the GGA-PBE approximation. The Murnaghan state equation has been employed to determine these lattice parameters, ensuring that the total energy of the investigated structures is minimized during the process. As the pressure increases from 0 GPa to 100 GPa with a 20 GPa step, the lattice parameters and unit cell volume of both compounds change. Detailed lattice parameters and cell volume of LiMCl_3_ compounds under varying pressures are listed in Table 1. Figs. 2(a) and (b) illustrate the decrease in lattice parameters and unit cell volume of both investigated compounds with increasing hydrostatic pressure, indicating a reduction in interatomic distances, respectively. This pressure-induced reduction in lattice parameters and unit cell volume has also been observed in previous studies^55,66,67^. The bond lengths of Li-Li, Li-Cl, Li-Mg (Be), and Mg (Be)-Cl decrease with increasing hydrostatic pressure, as shown in Figs. 2(c) and (d). Under increasing hydrostatic pressures, the repulsive forces between atoms become stronger, leading to an increase in the hardness of LiMCl_3_ (M = Mg, Be) perovskites. Under higher hydrostatic pressure, the interatomic spacing decreases, reducing the bond length and subsequently narrowing the energy gap between the valence band maximum (VBM) and conduction band minimum (CBM)^68,69^. The conduction band, which is more sensitive to changes in bond length than the valence band, shifts to lower energies at a faster rate, resulting in a noticeable bandgap decrease in LiMCl_3_ compounds. Due to the reduction in bond length, the repulsive interactions between atoms strengthen, increasing the resistance to compression under higher pressure conditions. Therefore, the relationship between bond length and hardness is inversely proportional; longer bonds result in weaker atomic attraction, while shorter bonds indicate stronger interactions. As this study shows the decreasing nature of bond length, which in turn leads to an increase in hardness, supported by the hardness values of this work. Similar effects have also been observed in previous studies^70^. Moreover, with the decrease in bond length, Poisson’s ratio of LiMCl_3_ increases, which also aligns with previously reported literature^71,72^. For experimental analysis, understanding the stability of the investigated LiMCl_3_ compounds is essential. As LiMCl_3_ are ABX_3_ type perovskite, we calculated both the Goldschmidt tolerance factor (t) and the octahedral factor ($\:\mu\:$) by the following formulas where X denotes the ionic radius of Cl, R_B_ denotes the ionic radius of Mg and Be, and R_A_ denotes the ionic radius of Li.

1 $$\:t=\frac{{R}_{A}+{R}_{X}}{\sqrt{2}\left({R}_{B}+{R}_{X}\right)}$$

2 $$\:\mu\:=\frac{{R}_{B}}{{R}_{X}}$$

Stability in a cubic structure requires the tolerance factor to fall within the range of 0.81 to 1.00, while the octahedral factor should be between 0.37 and 0.859. Our calculations confirm that both materials meet these criteria, ensuring their structural stability under ambient pressure. According to earlier research, the tolerance factor (t) can also be computed using the following alternate techniques, which use the bond lengths of Li-Cl, M (Mg, Be)-Cl.

3 $$\:\text{t}\:=\:\frac{0.707<Li-Cl>}{<M-Cl>}$$

According to earlier research, the structure is stable if the t value falls between 0.93 and 1.02^45^; Table 2 shows that our compounds under various hydrostatic pressures fall within this range.

**Table 1:** Lattice constants and unit cell volume of LiMCl_3_ under pressures.

Pressure (GPa)	Compounds	Lattice constants (Å)		Volume (Å^3^)
		This work	Other works	
0	LiMgCl_3_	4.96	4.95^56^	122.12
	LiBeCl_3_	4.56	4.56^56^	95.42
20	LiMgCl_3_	4.46	–	88.90
	LiBeCl_3_	4.18	–	73.28
40	LiMgCl_3_	4.24	–	76.71
	LiBeCl_3_	3.99	–	63.92
60	LiMgCl_3_	4.10	–	69.35
	LiBeCl_3_	3.87	–	58.08
80	LiMgCl_3_	4.00	–	64.16
	LiBeCl_3_	3.77	–	53.89
100	LiMgCl_3_	3.91	–	60.20
	LiBeCl_3_	3.70	–	50.65

**Table 2:** Goldschmidt tolerance factor (t) of LiMCl_3_ (M = mg, be) under hydrostatic pressures.

Pressure (GPa)	Tolerance factor	
	LiMgCl_3_	LiBeCl_3_
0	0.999	1.000
20	1.000	0.997
40	1.000	1.000
60	0.999	1.000
80	0.999	1.000
100	0.999	0.997

### Phonon dispersion

**Fig. 3:**
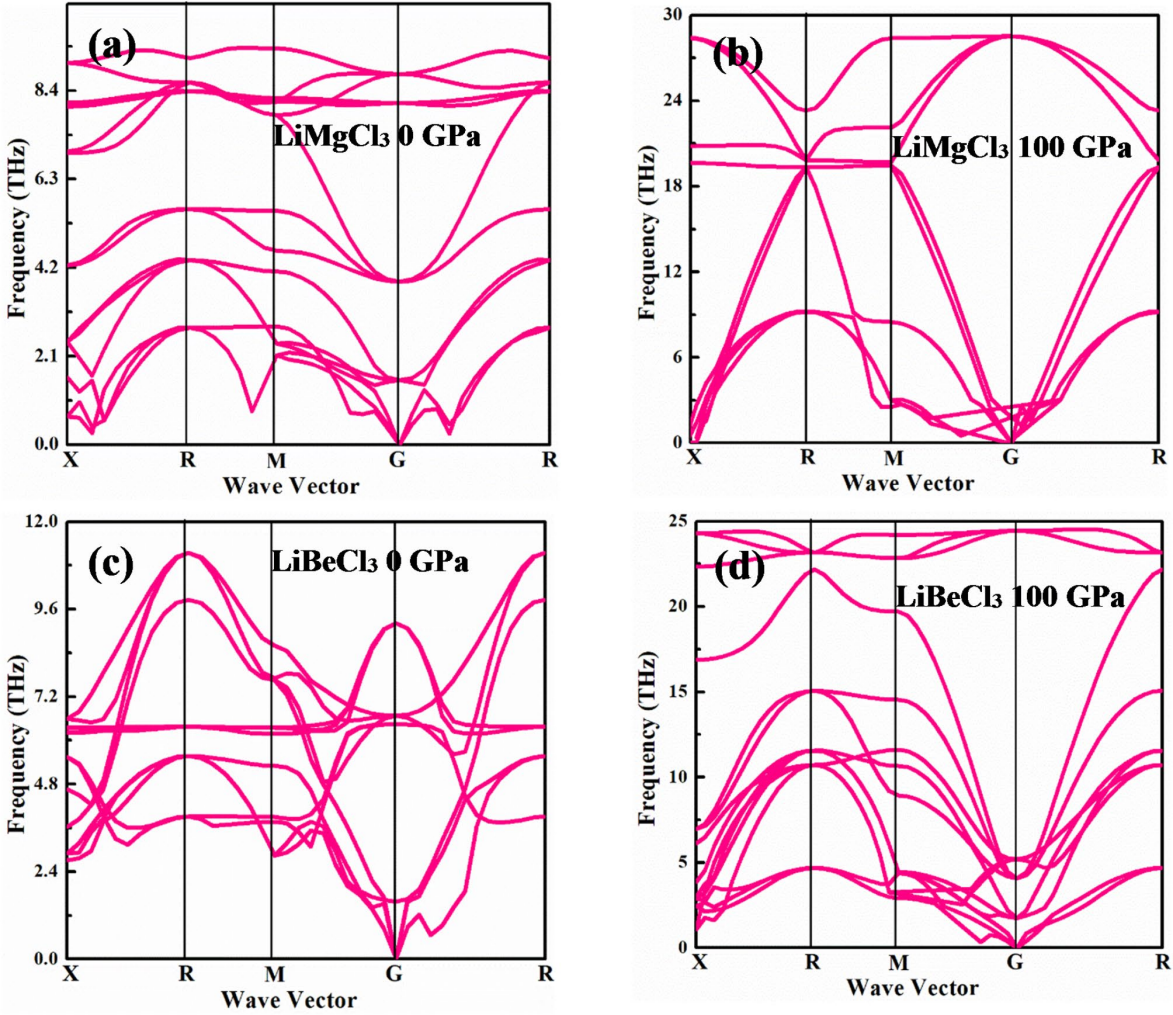
Phonon dispersion of LiMCl_3_ (M = Mg, Be) compounds for 0 GPa and 100 GPa pressure conditions.

The interaction between phonons’ momentum and energy in a crystal lattice is known as phonon dispersion. Quantized lattice vibration is known as phonons and their dispersion offers vital insights into the thermal behavior and vibrational characteristics associated with numerous materials^73^. Using a phonon dispersion curve, one can observe several bands that correspond to different frequencies of the crystal structure. In this study, phonon calculation has been employed to observe the stability of the investigated compounds under different pressure circumstances. It is evident that both materials with ambient pressure and higher hydrostatic pressure conditions vibrate in the positive mode, as can be seen from the computed phonon spectra in Fig. 3. The crystal lattice’s phonon dispersion curve displays positive frequencies that correspond to significant and physically acceptable vibrational modes that can be employed in optoelectronic devices.

### Electronic properties

**Fig. 4:**
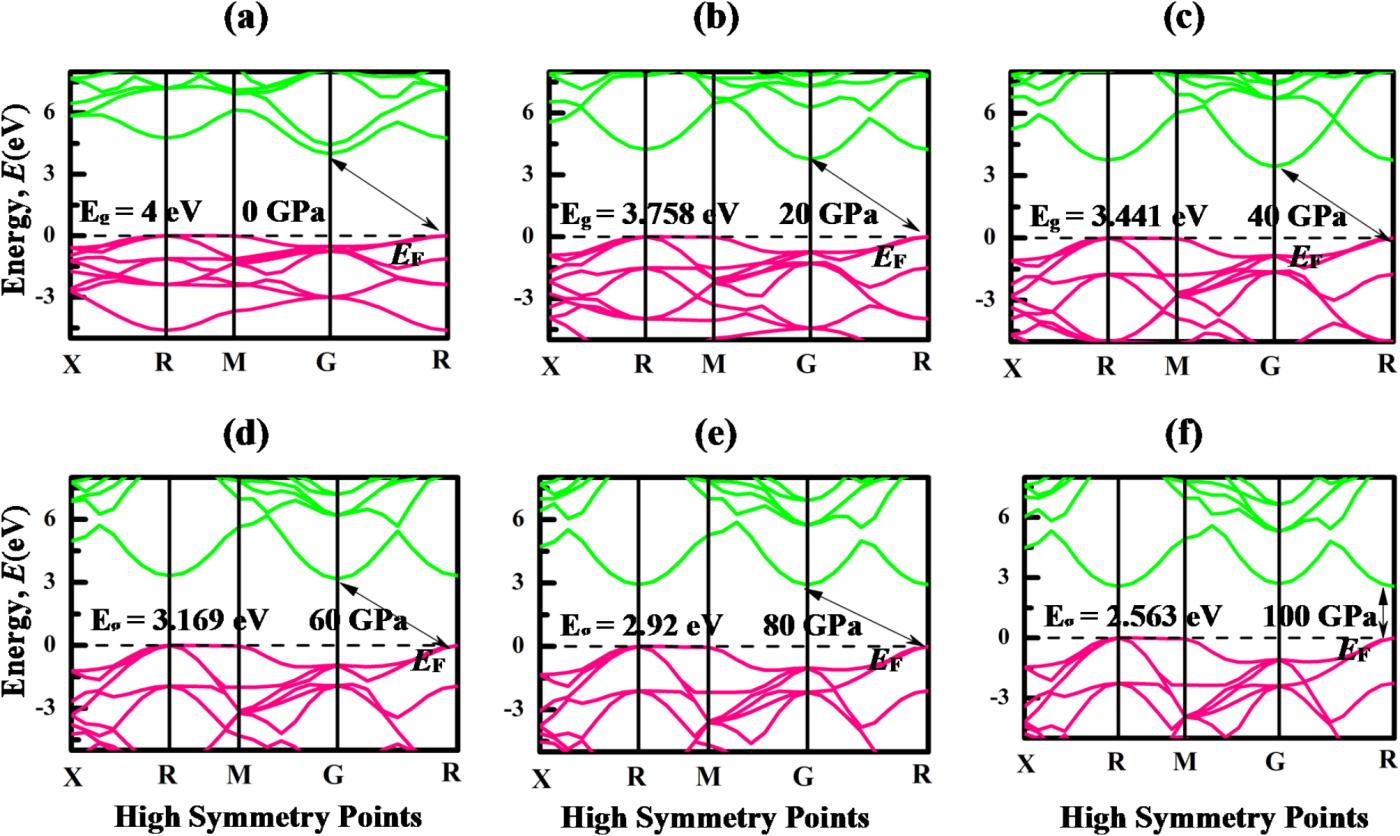
Band structures of LiMgCl_3_ under different hydrostatic pressure with GGA-PBE exchange-correlation functional.

**Fig. 5:**
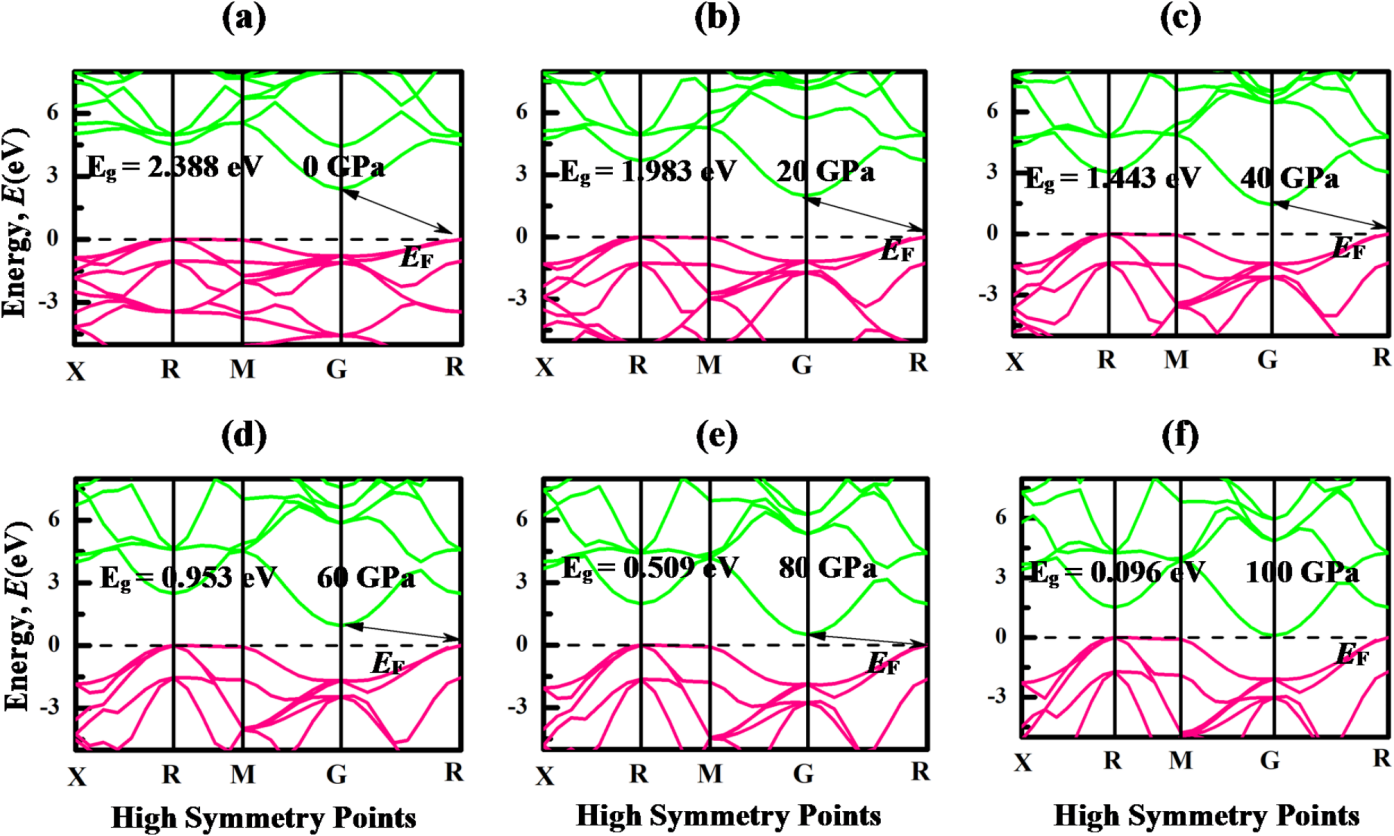
Band structures of LiBeCl_3_ under different hydrostatic pressures with GGA-PBE exchange-correlation functional.

The energy bands are a representation of the different energy levels that electrons can potentially occupy. Understanding the electronic band structure and Density of states (DOS) of a material is crucial for assessing the electronic behavior of a material. Band structure offers insights into the diverse energy levels and electronic states that consist in a material, while DOS elucidates the distribution of these energy states relative to energy. The electronic band structures of LiMCl_3_ along the high-symmetry points “X-R- M- G- R” in the first Brillion zone at various hydrostatic pressure ranges, from 0 to 100 GPa pressure with 20 GPa interval, are shown in Figs. 4 and 5. The energy range of the band structures spans from − 5 eV to + 8 eV, with the Fermi energy level positioned at 0 eV, marked by a black dotted line. The portion of the energy band situated above the Fermi level is known as the conduction band, while the segment below the Fermi level is termed the valence band. In this investigation, the GGA-PBE & HSE06 approximation has been considered to calculate the bandgap of the investigated compounds (Table 3). While some studies express concerns about the accuracy of GGA functional for bandgap calculations, it’s noteworthy that GGA functional don’t consistently underestimate the bandgap, as evidenced by several studies^74,75^. Under non-pressurized conditions, both LiMgCl_3_ and LiBeCl_3_ compounds exhibit CBM at G symmetry points and VBM at R symmetry points, resulting in an indirect bandgap of 4 eV for LiMgCl_3_ and 2.3 eV for LiBeCl_3_. The calculated bandgaps of these compounds closely align with other theoretical calculations, validating the accuracy of the GGA-PBE functional utilized in this study^56^. However, no experimental studies have been conducted on these materials to date. When hydrostatic pressure is applied, the CBM of LiMgCl_3_ compounds shifts towards lower energy levels along the G symmetry point. This shifting of energy band results in a decrease in bandgaps, providing significant insights into the inverse connection between bandgap and pressure, as supported by Fig. 4. This decrease in band gap can be attributed to a myriad of factors influenced by pressure, including alterations in bond angles, atomic configuration, and interatomic distance. Moreover, increased pressure also amplifies the potential between electrons and ions, leading to a reduction in lattice parameters and contribute to bandgap reduction. The transition from an indirect to a direct bandgap can be attributed to several factors, primarily the reduction in atomic distances within the crystal under higher pressure, which enhances orbital overlap between adjacent atoms. This increased overlap alters the electronic band structure, leading to changes in the energy levels of the conduction band minimum (CBM) and valence band maximum (VBM). In the LiMCl_3_ compounds, the CBM and VBM are initially located at different k-points, indicating a momentum mismatch. At higher hydrostatic pressure LiBeCl_3_ also shows the indirect bandgap with reduced bandgap energy. A significant reduction of energy gap is observed in this compound as depicted in Fig. 5, where its band gap experiences a notable decrease from 2.4 eV at 0 GPa to only 0.096 eV under 100 GPa pressure. This leads to speculation that over 100 GPa pressure may drive the compound toward a metallic state. This process reduces the distance between atoms in the LiBeCl_3_ compound resulting in a narrow band gap. By making it simpler to transfer electrons from VB to the CB, this shift in the band gap may increase the effectiveness of optoelectronic devices as reported by previous studies^76–78^. However, in indirect bandgap materials such as LiBeCl_3_, electron transitions require both photon absorption for energy transfer and phonon interaction to provide the necessary momentum change. LiMgCl_3_ also demonstrates a reduction in band gap under pressure, with a notable decrease from 4 eV to 2.563 eV at 100 GPa pressure (Table 3). However, based on the HSE06 calculation, the bandgap of LiMgCl₃ decreases from indirect 4.12 eV to direct 2.61 eV, while that of LiBeCl₃ decreases from indirect 3.71 eV to indirect 1.32 eV. Indirect bandgap materials are important in many devices, but direct bandgap materials are typically chosen for optoelectronic applications because of their effective light emission and absorption capabilities. Si-based devices also remain essential in infrared photodetectors, as highlighted by previous studies^79,80^. When exposed to a propagating far-field light source, silicon (Si), a commonly used indirect bandgap material, requires phonon assistance to excite carriers during photoexcitation which results in a decreased excitation efficiency. In order to overcome this constraint, scientists frequently use plasmonic resonance to increase the light field’s intensity^80^. Additionally, indirect bandgap semiconductors have demonstrated exceptional performance in thermoelectric applications, where energy conversion efficiency is maximized by an ideal bandgap that is usually a particular multiple of the operating temperature^81^.

**Table 3:** Bandgap energy (E_g_) of LiMgCl_3_ and LiBeCl_3_ compounds under pressures.

Pressure (GPa)	Compounds	Bandgap, E_g_ (eV)		Nature	Remarks
		GGA-PBE	HSE06		
0	LiMgCl_3_	4.0	4.12	Indirect	^56^
	LiBeCl_3_	2.39	3.71	Indirect	^56^
20	LiMgCl_3_	3.76	3.86	Indirect	This work
	LiBeCl_3_	1.98	3.28	Indirect	This work
40	LiMgCl_3_	3.44	3.57	Indirect	This work
	LiBeCl_3_	1.44	2.71	Indirect	This work
60	LiMgCl_3_	3.17	3.26	Indirect	This work
	LiBeCl_3_	0.95	2.20	Indirect	This work
80	LiMgCl_3_	2.92	3.00	Indirect	This work
	LiBeCl_3_	0.51	1.76	Indirect	This work
100	LiMgCl_3_	2.56	2.61	**Direct**	This work
	LiBeCl_3_	0.09	1.32	Indirect	This work

**Fig. 6:**
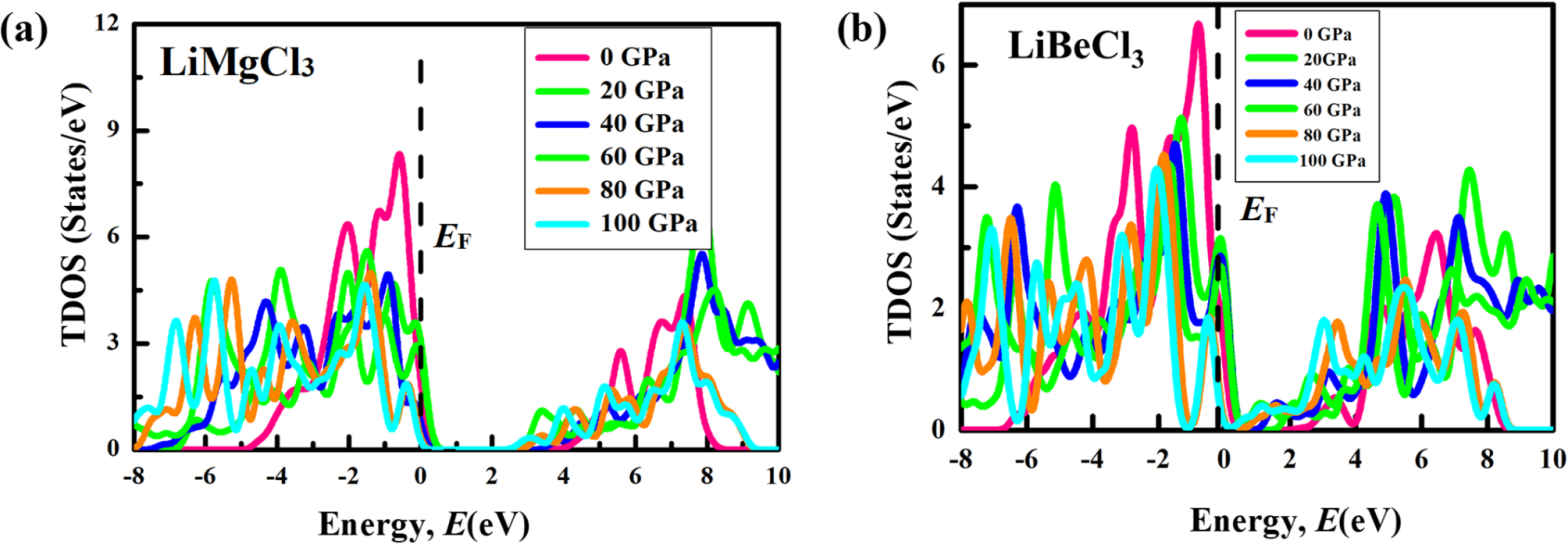
The total density of states (TDOS) of (**a**) LiMgCl_3_ and (**b**) LiBeCl_3_ compounds under hydrostatic pressures (0-100 GPa).

**Fig. 7:**
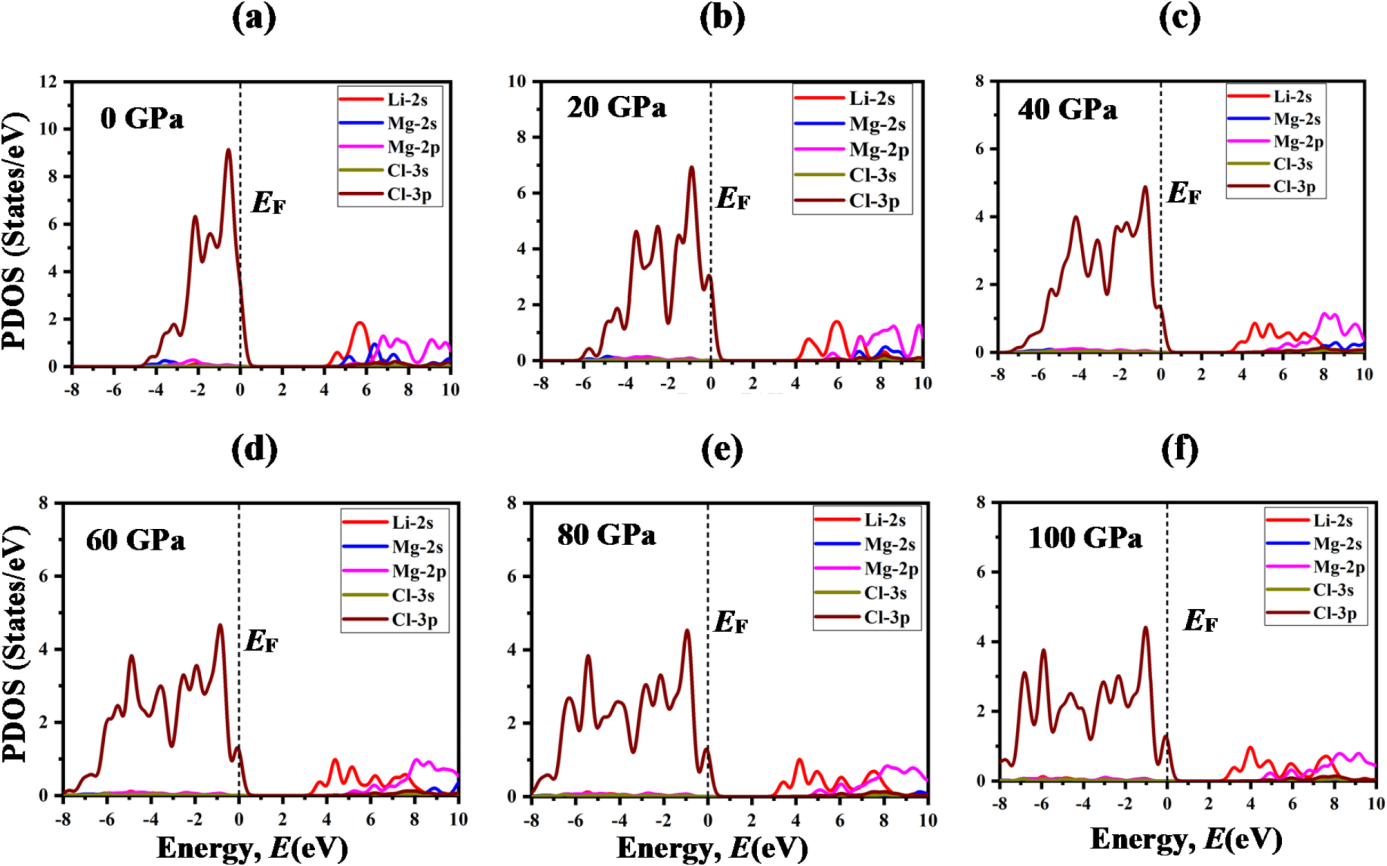
The partial density of states (PDOS) of LiMgCl_3_ under pressures (0-100 GPa).

**Fig. 8:**
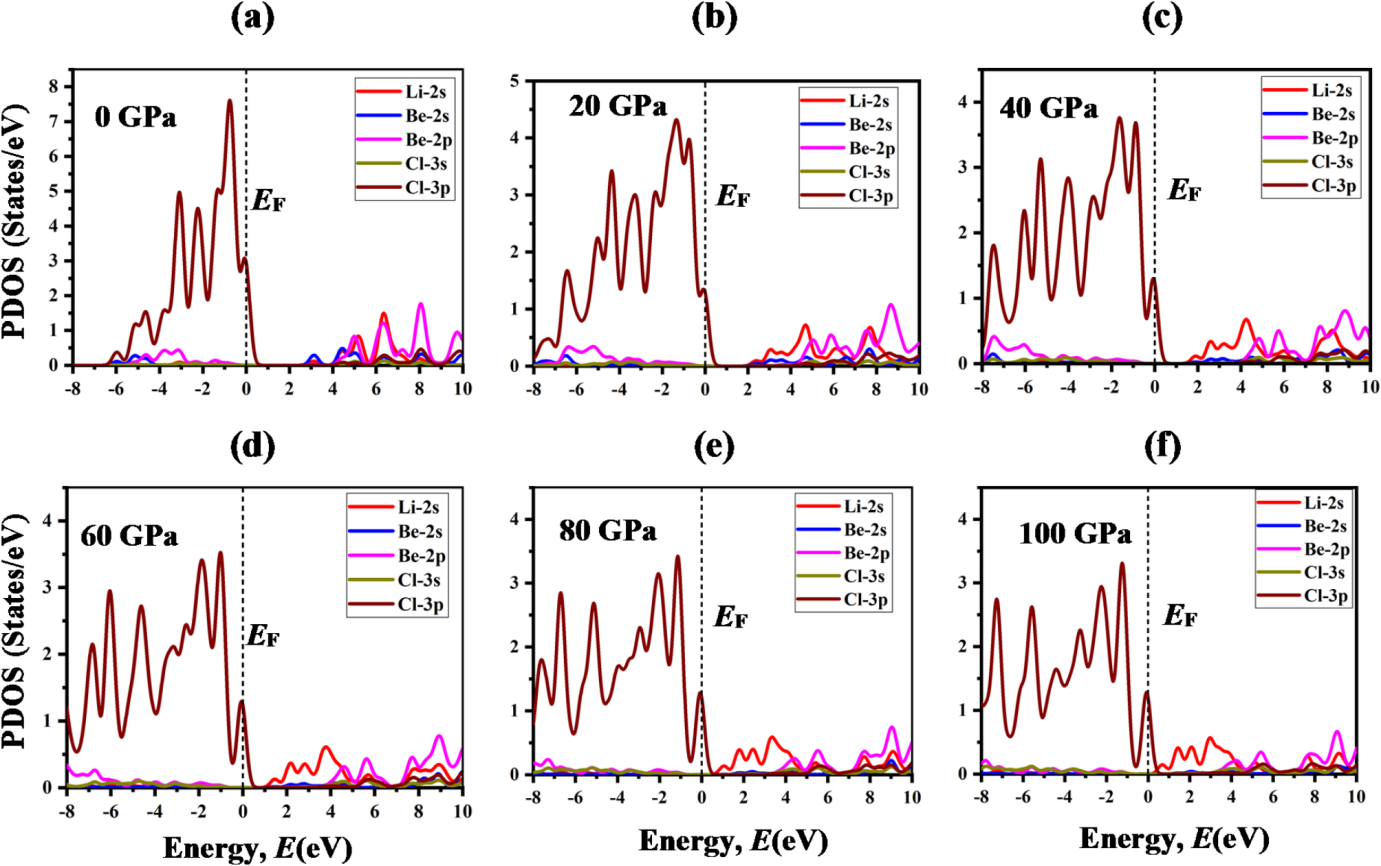
The partial density of states (PDOS) of LiBeCl_3_ under pressures (0-100 GPa).

To delve into the electronic band structure in-depth, total density of states (TDOS) and partial density of states (PDOS) is used to elucidate the contribution of different states to the formation of conduction and valance bands. The TDOS is the sum of all the states of each atom, and PDOS is the sum of all the contributions of each sub-state to the TDOS. Fig. 6 illustrates the TDOS of LiMCl_3_ compounds across an energy range from − 8 eV to + 10 eV under varying hydrostatic pressures. As pressure increases, notable variations in the TDOS become apparent which can be further elucidated by analyzing the PDOS as depicted in Figs. 7 and 8. For both LiMgCl_3_ and LiBeCl_3_ compounds, the TDOS below the fermi level known as valance band mostly originated from the Cl-3p state with a tiny contribution from the Mg-2p (Be-2p) state. TDOS above the Fermi level, known as the conduction band, is predominantly dominated by the Li-2s state, which plays a crucial role in bandgap reduction in high-pressure circumstances, comparable to the influence of Mg-2p (Be-2p) and Mg-2s (Be-2s) states. The hybridization of Li-2s and Mg-2s (Be-2s) also contributes to the reduction in bandgap. It’s noteworthy that the non-zero value of DOS at 0 eV underscores the metallic nature of LiMgCl_3_ at high pressure (100 GPa). In contrast, for LiBeCl_3_, a more pronounced metallic behavior has been observed at higher pressures (40–100 GPa) circumstances.

### Optical properties

The response of a material to electromagnetic radiation is crucial for assessing its potential in various industrial applications. This response is characterized by several energy/frequency-dependent parameters such as dielectric constant, absorption spectra, refractive index, reflectivity, and conductivity. To gain a comprehensive understanding, this section investigates the optical properties of LiMgCl_3_ and LiBeCl_3_ at different hydrostatic pressures (0-100 GPa) across an energy range of 0 eV to 30 eV.

The dielectric function ε(ω), which signifies a material’s linear response to an external electromagnetic field, offers valuable insights into its optical properties. The relationship between optical properties and the dielectric function, arising from light’s impact as it travels through materials, is mathematically expressed as ε(ω) = ε_1_(ω) + ε_2_(ω), with ε_1_(ω) and ε_2_(ω) representing the real and imaginary part of the dielectric function, respectively^82^.

**Fig. 9:**
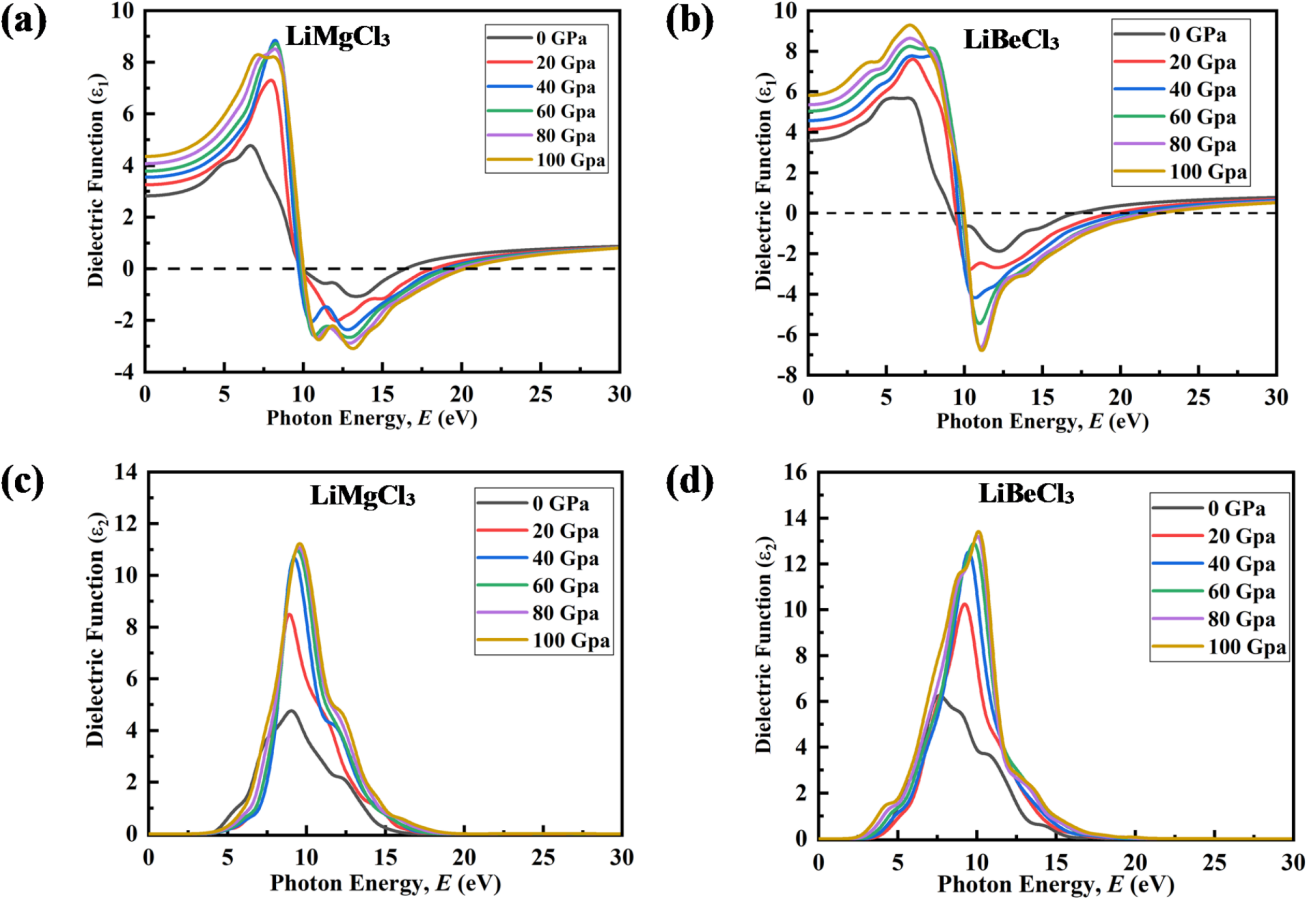
Pressure-induced variation of Real part of DF: (**a**) LiMgCl_3_, (**b**) LiBeCl_3_ and Imaginary part of DF: (**c**) LiMgCl_3_, (**d**) LiBeCl_3_.

In this study, the dielectric function ε(ω) of LiMCl_3_ (M = Mg, Be) is illustrated in Fig. 9. The real component of the dielectric function (RDF) serves as an efficient parameter, offering crucial information about the charge carrier recombination rate and thereby the overall effectiveness of optoelectronic devices^83^. Moreover, it is evident from the RDF that the spectral patterns of the two materials are consistent with each other.

At ambient pressure, the static dielectric constant ε_1_(0) of LiMgCl_3_ is measured to be 2.82, which increases to 4.35 at 100 GPa pressure, while LiBeCl_3_ shows ε_1_(0) of 3.58 at ambient pressure, increasing to 5.82 under 100 GPa pressure as shown in Fig. 9(a) and (b). Materials with a substantial band gap typically exhibit a low static dielectric constant. The ε_1_(0) value increases with induced pressure for both compounds, correlating with a decrease in band gap under pressures. This decrease in band gap ensures a greater number of electrons can transfer from valence bands (VBs) to conduction bands (CBs), thereby reducing the recombination rate^52^. Additionally, both LiMgCl_3_ and LiBeCl_3_ exhibit negative ε_1_(ω) values within an energy range from 9.2 eV to 18 eV and 10 eV to 18 eV, respectively, suggesting drude-like characteristic of these compounds, confirming their increasing metallic nature under high-pressure circumstances^84^.

**Fig. 10:**
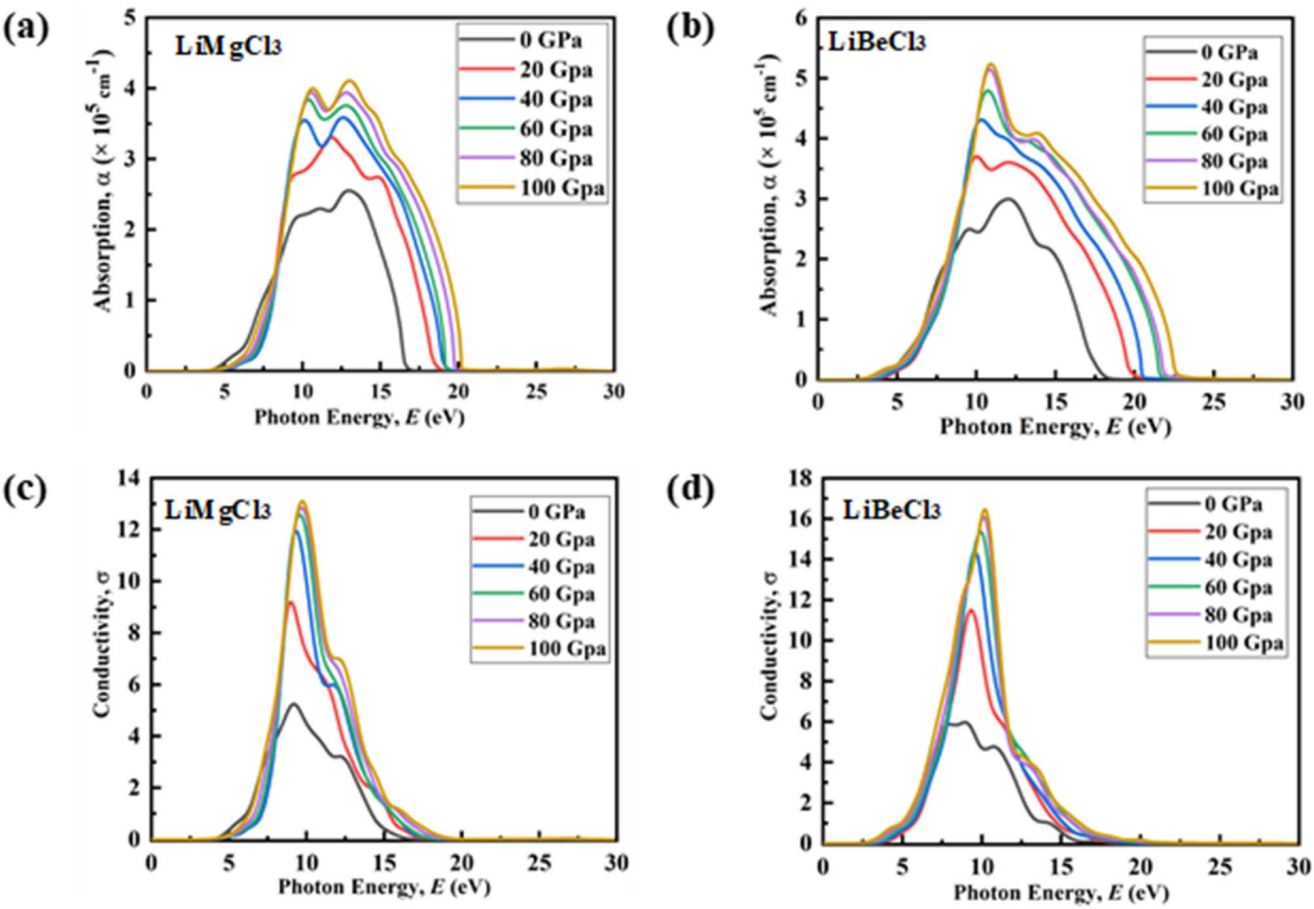
Pressure-induced variation of Absorption coefficient: (**a**) LiMgCl_3_, (**b**) LiBeCl_3_ and Conductivity: (**c**) LiMgCl_3_, (**d**) LiBeCl_3_.

The imaginary component of the dielectric function (IDF) plays a crucial role in elucidating the absorption behavior of materials^85^. It is directly influenced by the band structure and DOS of a material. As depicted in Figs. 9(c) and (d), the peak of ε_2_(ω) for LiMCl_3_ (M = Mg, Be) compounds increases as pressure rises. This behavior is in agreement with the absorption spectra depicted in Figs. 10(a) and (b). Additionally, these peaks shift towards higher energies with increased pressure, attributed to the widening of the valence band and the downward shift of the lower valence band^86^. ε_2_(ω) tends to decrease from its peak value with increasing photon energy, eventually reaching zero. The distinctive pattern of higher ε_2_(ω) values at lower frequencies and smaller ε_2_(ω) values in the higher energy range highlights the potential applicability of LiMCl_3_ (X = Mg, Be) in microelectronics and integrated circuits. This superiority is notably amplified under pressure, indicating the material’s enhanced performance characteristics in such applications^75^.

Studying the absorption coefficient (α) is pivotal in understanding how a material interacts with specific photon energies, influencing the attenuation of light^87^. This investigation provides valuable insights into a material’s compatibility and potential for enhanced performance in various device applications. The absorption coefficient variations of LiMgCl_3_ and LiBeCl_3_ across different hydrostatic pressures are depicted in Figs. 10(a) and (b). In both materials, absorption does not begin at 0 eV due to the presence of optical bandgap. At ambient pressure, absorption starts at about 2.391 eV and 3.978 eV of photon energy for LiBeCl_3_ and LiMgCl_3_ respectively, signifying their specific optical bandgap energies. For both compounds, a major absorption peak has been observed within a broad region of photon energy. By applying pressure, the absorption spectrum for both compounds becomes narrower, heightened, and broadened, falling within the ultraviolet region. This indicates the potentiality of LiMgCl_3_ and LiBeCl_3_ compounds in fabricating devices for sterilizing surgical equipment^88^. These two materials demonstrate higher absorption in the UV region, with their absorption rate increasing as hydrostatic pressure rises, despite a decrease in their bandgap under incremental pressure. This behavior underscores the motivation to apply higher hydrostatic pressure to these materials. These materials are suitable for use in tandem solar cells, where each layer is designed to absorb different parts of the solar spectrum. These materials can be used as the top layer to absorb high-energy UV photons, while other layers are optimized for absorbing visible and infrared light. This configuration helps reduce thermal losses, resulting in more efficient utilization of these materials. Under high pressure, LiMgCl_3_ exhibits a direct bandgap, while LiBeCl_3_ shows an indirect bandgap under varying pressure conditions. As a result, LiMgCl_3_ is likely to be more efficient in these conditions, as electron transitions in direct bandgap materials do not require phonon assistance, eliminating the momentum mismatch between the valence and conduction bands.

The optical conductivity (σ) stands as a fundamental parameter that characterizes a material’s electromagnetic response^89^. Figs. 10(c) and (d) depict the real part of conductivity under various hydrostatic pressures (0–100 GPa). It is observed for both materials that σ does not initiate at 0 eV. This may be attributed to the specific optical bandgap of these materials under different hydrostatic pressures. However, beyond their optical bandgap, both materials smoothly reach their peaks under different hydrostatic pressures. The figures clearly show that the optical conductivity enhances with increasing pressure, correlating with the heightened absorption coefficient under pressures. This enhancement in conductivity is attributed to electron transitions from the valence to the conduction band triggered by light absorption^53^. Intriguingly, the peaks in σ become sharper under hydrostatic pressure. For LiMgCl_3_, the conductivity peak shifts from 9.18 eV at zero pressure to 9.83 eV at 100 GPa, while for LiBeCl_3_, the peak shifts from 8.9 eV to 10.15 eV under similar pressure conditions. These shifts align with the pressure-induced changes in the band structure, characterized by a narrowing band gap with increasing pressure^90^.

**Fig. 11:**
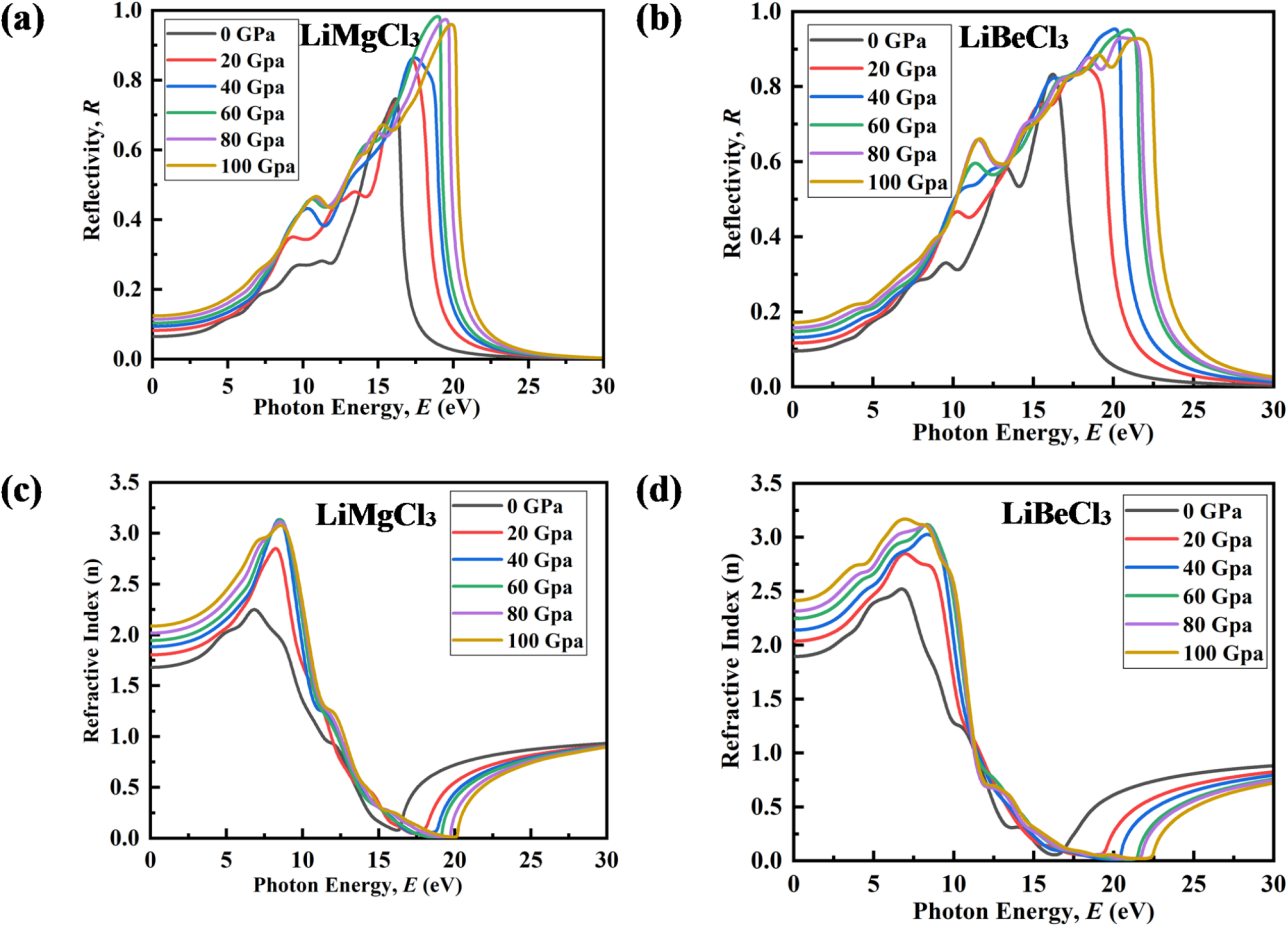
Pressure-induced variation of Reflectivity: (**a**) LiMgCl_3_, (**b**) LiBeCl_3_ and Refractive index: (c) LiMgCl_3_, (**d**) LiBeCl_3_.

The reflectivity spectra (R) of LiMgCl_3_ and LiBeCl_3_ under varying pressures are depicted in Figs. 11(a) and (b). R is a key optical parameter, indicating a material’s ability to reflect light energy, and is inversely related to its light absorption^91^. Both materials show similar trends in their reflectivity spectra. At lower photon energies, the reflectivity remains relatively low and experiences minimal changes with increasing pressure. However, within the energy range of 10 eV to 20 eV, a noticeable variation in reflectivity has appeared under pressure. This observation implies that LiMgCl_3_ and LiBeCl_3_ can be effectively controlled using external pressure, making them suitable coating materials within this energy range^92^.

The refractive index, a dimensionless parameter indicating electromagnetic wave speed in a material, is illustrated in Fig. 11(c) and (d) across the energy of 0 eV to 100 eV and pressures from 0 to 100 GPa. Analysis of the figures reveals that, at ambient pressure, normal dispersion is observed between 0 eV – 7.5 eV and 16.3 eV – 30 eV, while abnormal dispersion is noted in the 7.5 eV-16.3 eV photon energy range. Additionally, LiBeCl_3_ exhibits a consistently higher maximum refractive index compared to LiMgCl_3_ at 0 GPa pressure. This difference signifies a stronger electronic polarization effect in LiBeCl_3_, leading to a greater delay in light propagation. When applying pressure, both materials display an increase in refractive index, reaching a maximum of around 10 eV due to the growing number of electronic oscillators per unit volume^93^. However, at a photon energy of 11.35 eV, the refractive index falls below unity for both materials, regardless of pressure, due to the group velocity of incident radiation surpassing the speed of light^94^. This indicates that the investigated materials transform into a superluminal medium when subjected to high-energy photons.

### Mechanical properties

Elastic constants (*C*_*ij*_) are crucial parameters for understanding the mechanical properties of a material, as they provide valuable insights into the material’s stability and stiffness^95,96^. By analyzing the *C*_*ij*_ values, one can determine a crystal’s ability to resist external forces, thereby gaining practical information about its potential applications. Thermal expansion, Gruneisen factor, specific heat, and Debye temperature are among the thermodynamic properties closely intertwined with elastic constants. These parameters also offer valuable insights into elastic properties such as thermo-elastic stress, internal strain, load deflection, sound velocity, and fracture toughness^66^. To calculate *C*_*ij*_, the finite strain theory has been used^66^. There are three independent elastic constants for a cubic phase of a compound including, longitudinal compression (C_11_), shear modulus (C_44_), and transverse expansion (C_12_). Under varying pressures, a material’s lattice parameters undergo alterations, accentuating the necessity to calculate elastic constants across various pressure conditions. This study also delves into the determination of pressure-induced elastic constants for LiMCl_3_, aiming to discern changes in elastic constants and mechanical properties. Table 4 presents the elastic constant values under distinct hydrostatic pressures. The stability of the cubic structure is evaluated using the Born stability criteria^97–99^: (C_11_ + 2C_12_) > 0, C_44_ > 0, and (C_11_ – C_12_) > 0. However, the Cij values of both investigated compounds satisfy these criteria, thereby confirming the stability of these compounds under varying hydrostatic pressures.

The mechanical robustness of different optoelectronic devices under thermal cycles is greatly influenced by the stiffness of the material, which should be carefully chosen to prevent mechanical delamination. Based on Young’s and bulk modulus values, the stiffness of the studied LiMCl_3_ compounds increases with rising hydrostatic pressure. Higher stiffness reduces deformation, which can improve the structural integrity of the device by reducing cracks and delamination, two main failure modes in optoelectronic devices^100^.

**Table 4:** Elastic constants (*C*_*ij*_) of LiMCl_3_ under different hydrostatic pressures.

Pressure (GPa)	Compounds	C_11_	C_44_	C_12_	C_12_-C_44_
0	LiMgCl_3_	60.48	12.71	18.19	5.48
	LiBeCl_3_	51.92	30.53	38.83	8.30
20	LiMgCl_3_	200.02	19.49	57.77	38.28
	LiBeCl_3_	163.36	55.55	94.30	38.75
40	LiMgCl_3_	320.00	24.55	93.50	68.95
	LiBeCl_3_	256.18	76.49	140.93	64.44
60	LiMgCl_3_	432.96	27.40	126.85	99.45
	LiBeCl_3_	347.75	95.97	187.78	91.81
80	LiMgCl_3_	540.75	30.82	160.32	129.50
	LiBeCl_3_	429.75	114.48	228.80	114.32
100	LiMgCl_3_	644.46	34.09	192.43	158.34
	LiBeCl_3_	509.72	132.01	271.08	139.07

Cauchy pressure is used to determine the ductility or brittleness of a material based on its positive or negative values^101^. A positive value of Cauchy pressure indicates that the material is ductile, while a negative value signifies that the material is brittle. The investigated compounds exhibit positive Cauchy pressure values under different hydrostatic pressures, confirming their ductile behavior. As the pressure increases, the positive Cauchy pressure values become even more positive, highlighting the increased ductility of the LiMCl_3_ (M = Mg, Be) compounds.

The bulk modulus and shear modulus are calculated using the Voigt^102^, Reuss^103^, and Hill approximations^104^, with the Hill approximation often considered the final value due to its closer alignment with experimental results, as supported by several studies. The bulk modulus, Young’s modulus, and shear modulus using the Voigt, Reuss approximations can be determined from the following relationships^105,106^:

4 $$\:B=\frac{{B}_{v}+{B}_{R}}{2}$$

5 $$\:G=\frac{{G}_{v}+{G}_{R}}{2}$$

6 $$\:E=\frac{9BG}{3B+G}$$

Mechanical properties, such as bulk modulus, Young’s modulus, Poisson’s ratio, and Pugh’s ratio, determined in this study are summarized in Table 5. It’s clear from the data that as pressure escalates, the values of bulk modulus (B), Poisson’s ratio (ν), and shear modulus (G) also increase. This trend signifies a rise in the stiffness and hardness of LiMCl_3_ (M = Mg, Be) compounds under increasing pressures.

**Table 5:** Calculated bulk modulus B (GPa), shear modulus G (GPa), Young’s modulus E (GPa), Poisson’s ratio ($\:\nu\:$), Pugh’s ratio (B/G), Zener anisotropy (A), Universal anisotropy factor (A^U^), Hardness ($\:{H}_{v}$), and Machinability ($\:{\mu\:}_{M}$) of LiMCl_3_ under pressure.

Pressure (GPa)	Compounds	B	G	B/G	E	$\:\nu\:$	A	A^U^	$\:{H}_{\nu\:}$	$\:{\mu\:}_{M}$
0	LiMgCl_3_	32.289	15.609	2.06	40.33	0.291	0.601	0.316	2.17	2.53
	LiBeCl_3_	43.198	16.659	2.59	44.284	0.329	4.667	3.458	1.89	1.41
20	LiMgCl_3_	105.191	33.813	3.11	91.622	0.354	0.274	2.306	3.29	5.39
	LiBeCl_3_	117.32	45.9	2.55	121.83	0.326	1.608	0.276	5.328	2.11
40	LiMgCl_3_	169.002	47.897	3.52	131.288	0.37	0.216	3.394	4.15	6.88
	LiBeCl_3_	179.35	68.29	2.62	181.8	0.331	1.327	0.096	7.69	2.34
60	LiMgCl_3_	228.89	59.236	3.86	163.596	0.38	0.179	4.516	4.74	8.35
	LiBeCl_3_	241.11	89.22	2.7	238.27	0.335	1.199	0.039	9.81	2.51
80	LiMgCl_3_	287.135	70.476	4.07	195.438	0.386	0.162	5.198	5.35	9.31
	LiBeCl_3_	295.79	108.65	2.72	290.41	0.336	1.139	0.02	11.88	2.58
100	LiMgCl_3_	343.114	81.244	4.22	225.903	0.390	0.15	5.736	5.95	10.06
	LiBeCl_3_	350.63	126.78	2.76	339.43	0.338	1.106	0.012	13.69	2.65

**Fig. 12:**
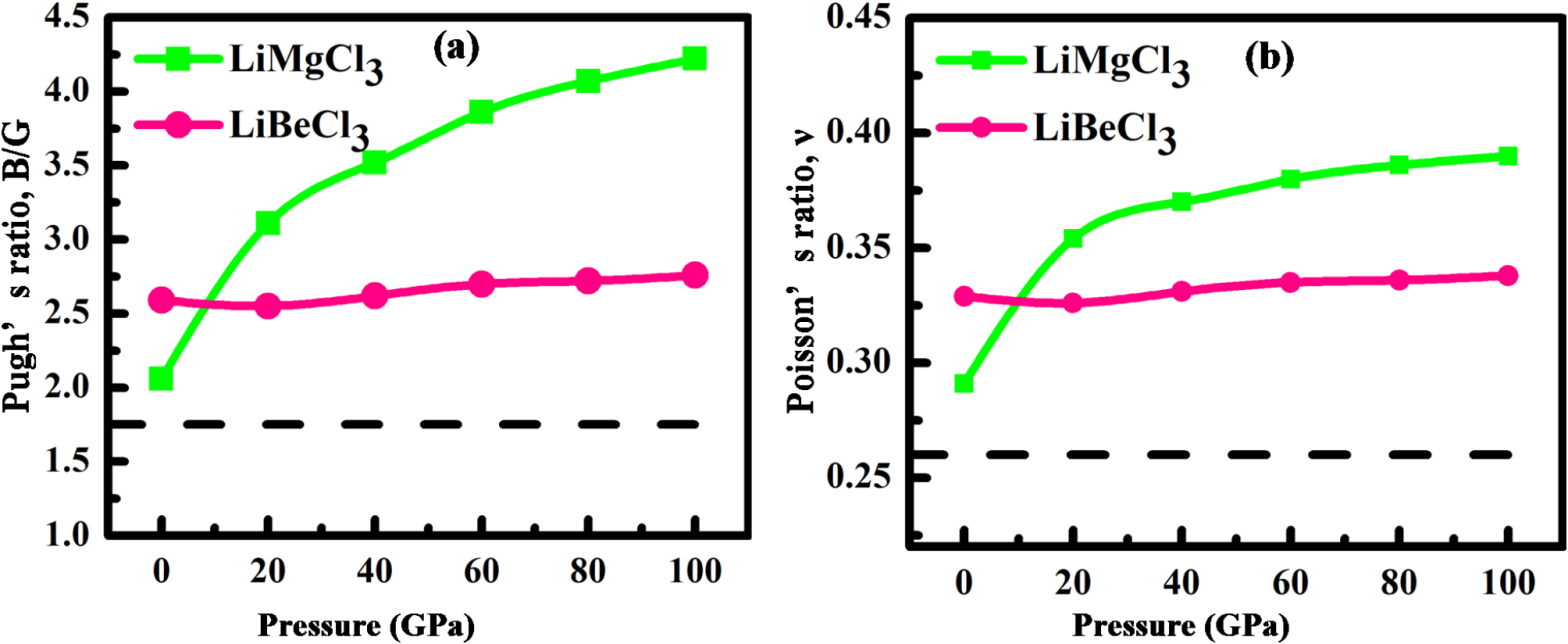
(**a**) Pugh’s ratio and (**b**) Poisson ratio of LiMgCl_3_ and LiBeCl_3_ under pressures.

Poisson’s and Pugh’s ratios are calculated as well in this study to provide precise insights into a material’s brittleness or ductility. According to Table 5, the threshold value for Poisson’s ratio is 0.26; above this value, a material is considered ductile, while below it, the material is considered brittle. The investigated compounds in this study exhibit ductile behavior under varying hydrostatic pressures. Moreover, Poisson’s ratio provides information on whether a material’s bonding force is central or non-central^107^. A central force is directed toward a fixed point and its magnitude depends on the distance of the particle from that point. In contrast, a non-central force does not simply depend on the distance between the centers of two interacting bodies^108^. Bonding in solids is considered central when the Poisson’s ratio is between 0.25 and 0.50. This study’s findings confirm that the LiMCl_3_ (M = Mg, Be) compounds exhibit central force characteristics. Similarly, to Poisson’s ratio, Pugh’s ratio (B/G) serves as a critical indicator for discerning the ductility and brittleness of a material. The materials above the threshold value of 1.26 are considered ductile, while those below are brittle. Figs. 12(a) and (b) visually presented the variations in Poisson’s ratio and Pugh’s ratio respectively under different hydrostatic pressures. Notably, at ambient pressure, the materials exhibit ductile behavior, with ductility further increasing as pressure rises which is also supported by the analysis of Poisson’s ratio and Cauchy pressure, proofing the accuracy of this study. This observation aligns with the analyses of Poisson’s ratio and Cauchy pressure, validating the accuracy and robustness of this study.

The machinability index ($\:{\mu\:}_{M}$) stands as a crucial mechanical property, defining the ease or difficulty with which a material can be machined using a cutting tool. It closely associates with both the bulk modulus and the C_44_ elastic constant, as delineated by the following equation:

7 $$\:{\mu\:}_{M}=\frac{B}{{C}_{44}}$$

Machinability holds paramount importance for materials that are earmarked for industrial applications. It governs various factors, including the selection of cutting tools, the optimal cutting speed, and the geometry of the cutting tool, etc. Enhanced machinability results in heightened lubricating properties, diminished friction values, and higher plastic strain. Pressure-induced variations in machinability have been investigated in this study and are presented in Table 5. A minor variation in the machinability index has been observed under increasing hydrostatic pressure for the LiBeCl_3_ compound. Conversely, higher $\:{\mu\:}_{M}$ values are observed for LiMgCl_3_ under high-pressure circumstances, suggesting exceptional lubricating properties of this compound.

Vickers hardness is indeed a crucial mechanical property, reflecting a material’s resistance to deformation. Understanding a material’s elastic and plastic behaviors significantly depends on knowing its hardness value. There’s a close relationship between hardness and Poisson’s ratio, which is established by the following relation^109^:

8 $$\:{H}_{\nu\:}=\frac{\left(1-2\nu\:\right)E}{6\left(1+\nu\:\right)}$$

Table 5 suggests that the hardness of LiMCl_3_ (M = Mg, Be) compounds typically increases with rising pressure. Notably, LiMgCl_3_ shows a significant increase under high-pressure conditions. In contrast, the hardness LiBeCl_3_ demonstrates a comparatively milder increasing trend, suggesting lower hardness compared to LiMgCl_3_.

**Fig. 13:**
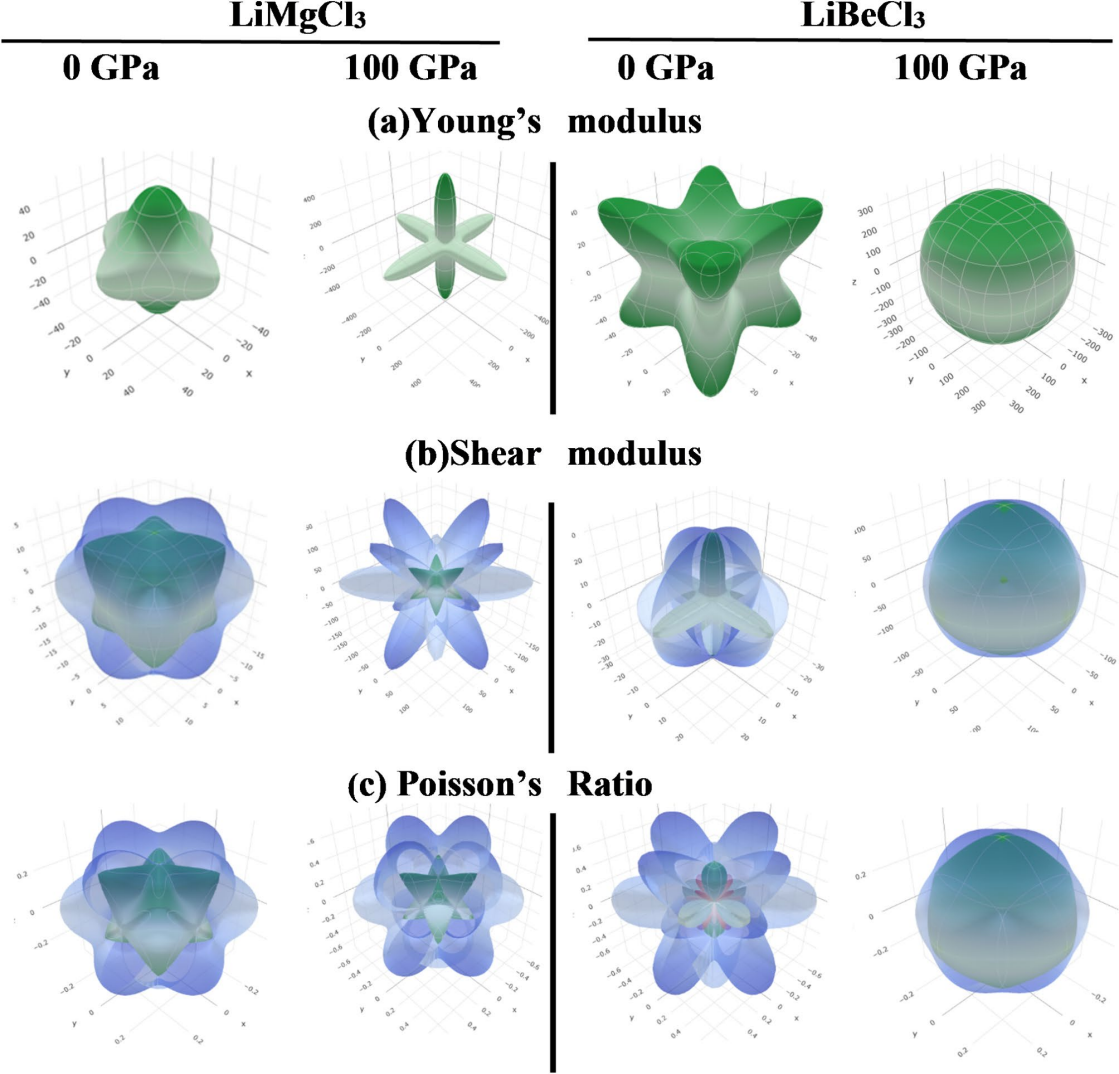
3D anisotropic representation of (**a**) Young’s modulus, (**b**) shear modulus, and (**c**) Poisson’s ratio of LiMgCl_3_ and LiBeCl_3_ under 0 GPa and 100 GPa pressures.

The anisotropy factor of a crystal is crucial as it strongly correlates with the likelihood of inducing microcracks in materials. The performance of a compound in a particular technological application depends significantly on its level of anisotropy^110^. As the investigated compounds possess cubic symmetry, A_1_, A_2_, and A_3_ are equal. The anisotropy of a material can be evaluated using its elastic constants through the following relation^111,112^:

9 $$\:A=\frac{2{C}_{44}}{{C}_{11}-{C}_{12}}$$

When the anisotropic factor value is 1, a material is considered isotropic, while deviation from this unity value indicates the degree of anisotropy present in the material. Anisotropic factor values of LiMCl_3_ under hydrostatic pressures are presented in Table 5. It is evident that the degree of anisotropy increases with pressure for LiMgCl_3_, whereas the opposite trend has been noticed in LiBeCl_3_ where the value of the anisotropy factor tends to approach towards unity as pressure rises. To provide a clearer visualization, Fig. 13 presents the 3D contour plots, illustrating the directional dependence of the anisotropy factor on Young’s modulus, shear modulus, and Poisson’s ratio for LiMCl_3_ (M = Mg, Be) compounds under pressures of 0 GPa and 100 GPa. Typically, an isotropic material displays a spherical shape in these plots. However, any deviations from this spherical shape indicate the degree of anisotropy of a material. For LiMgCl_3_, a significant deviation from the spherical shape is observed at 100 GPa, indicating a higher degree of anisotropy. In contrast, LiBeCl_3_ shows a more spherical shape at 100 GPa, implying that this compound becomes more isotropic under high-pressure circumstances.

To further elucidate the anisotropy nature of LiMCl_3, the_ universal anisotropy factor ($\:{A}^{U}$) has been used. It can also be obtained through the following relation^113^:

10 $$\:{A}^{U}=5\left(\frac{{G}_{v}}{{G}_{R}}-1\right)$$

The value of $\:{A}^{U}$ for isotropic compounds is zero whereas any deviation from this value indicates the degree of anisotropy. Pressure-induced $\:{A}^{U}$ for different hydrostatic pressure has been calculated and listed in Table 5. It is evident that LiMgCl_3_ exhibits increased anisotropy under rising hydrostatic pressures, whereas LiBeCl_3_ becomes more isotropic under higher pressure conditions. However, these observations are also supported by Fig. 12.

## Conclusion

In summary, the physical properties of the investigated compounds LiMgCl_3_ and LiBeCl_3_ change under varying hydrostatic pressure, ranging from 0 GPa to 100 GPa. The lattice parameters and unit cell volume decrease with increasing pressure, likely due to the reduction in bond length between constituent atoms. The electronic properties, including band structure, TDOS, and PDOS, are also examined under different pressure conditions. LiMgCl_3_ exhibits a transition from an ultrawide bandgap to a narrow bandgap, while LiBeCl_3_ transitions from a semiconductor to a metallic state under 100 GPa pressure. This reduction in energy bandgap enhances the efficiency of these materials in optoelectronic applications. Improved dielectric function, higher reflectivity, and refractivity are observed under high-pressure conditions, with absorption peaks shifting upwards in the UV region, indicating their potential use in optoelectronic devices. Additionally, the elastic constants of both materials suggest ductile behavior, which increases with applied pressure. The anisotropy factor shows an increasing trend for LiMgCl_3_ with rising pressure, while it decreases for LiBeCl_3_, indicating different anisotropic behaviors under pressure. This study highlights the potential application of alkali earth metal-based perovskites, particularly Li-based compounds, in future optoelectronic device applications.

## Data Availability

The data used and/or analyzed during the current study are available from the corresponding author upon reasonable request.
